# Smart nanoplatforms for early detection and immune modulation in lung cancer

**DOI:** 10.3389/fbioe.2025.1734570

**Published:** 2026-01-22

**Authors:** Chaoxiao Yu, Haiying Xia, Yanqing Wang, Xueping Liu

**Affiliations:** 1 Department of Pulmonary and Critical Care Medicine,Yantaishan Hospital, Yantai, China; 2 Emergency Intensive Care Unit, The Affiliated Taian City Central Hospital of Qingdao University, Taian, China; 3 Department of Pulmonary and Critical Care Medicine, Haiyang People’s Hospital, Haiyang, China; 4 Department of Pulmonary and Critical Care Medicine, Yantai Yuhuangding Hospital, Yantai, China

**Keywords:** artificial intelligence, early cancer detection, immune modulation, lung cancer, nanotechnology

## Abstract

Lung cancer is a major cause of cancer related mortality due to delayed diagnosis and limited therapeutic efficiency. Early detection and effective immune modulation are important to control lung cancer. Advancements in nanotechnology have improved oncology due to sensitive, specific, and minimally invasive detection platforms along with immune regulatory therapeutic approaches. Smart nanoplatforms fabricated with high precision and responsiveness have the ability to treat diseases as well as the immune system. These systems combine functional nanomaterials with biomolecular recognition elements to detect biomarkers such as exosomes, DNA, RNA, and proteins. They also facilitate targeted immune activation through checkpoint inhibition, nanovaccines, and tumor microenvironment reprogramming. Moreover, artificial intelligence and machine learning are enhancing the interpretation of complex data, which increases the diagnostic accuracy and predictive power. Despite advances in diagnostic and immune modulation, there are also several challenges related to biological barriers and biocompatibility. This review comprehensively explains the molecular basis of lung cancer, recent progress in nanotechnology based diagnostics and immunotherapy, and the design of multifunctional smart nanoplatforms. Future studies emphasize integrating personalized medicine, digital modeling, and bioinspired nanosystems for clinically translatable solutions in early lung cancer management.

## 1 Introduction

Cancer is a leading cause of death throughout the world, posing a significant threat to human life and health (Kumbrink et al., 2024; Guo C. et al., 2025). Lung cancer is one of the most challenging global health issues (Nokes et al., 2023; Chen R.-K. et al., 2025). Main risk factors for lung cancer and other pulmonary disorders include tobacco use, ambient and household air pollution, asbestos exposure, and second-hand smoke continue to drive the high burden, especially in low and lower-middle-income countries, where regulatory resources and access to healthcare are more limited (Wang et al., 2024a; Wang et al., 2024b). Household air pollution from solid fuels has been shown to significantly contribute to tracheal, bronchial, and lung cancer (TBL) among people aged 55 and above (He et al., 2025; Xing et al., 2025). Early detection is the single most important element of survival (Chen Y.-N. et al., 2025). Yet, most patients are still diagnosed at last stages because population screening is limited, symptoms are nonspecific, and existing screening tools face cost, accessibility, and false positive limitations (Nooreldeen and Bach, 2021; Gasparri et al., 2023). At the molecular level, lung cancers are highly heterogeneous, driven by diverse genomic alterations and a suppressive tumor microenvironment that together complicate accurate early diagnosis and effective, durable therapy (Li et al., 2023). Immunotherapies have transformed care for subsets of patients, but primary and acquired resistance, variable biomarker performance, and immune related toxicities limit population level impact (Rother et al., 2024; Liu et al., 2025). Moreover, increasing environmental pollution is also a cause of lung complications in nonsmokers (Luo J. et al., 2025). Due to these clinical and epidemiologic challenges, there is an urgent need to develop sensitive, specific, and accessible approaches that can detect lung cancer earlier and safely modulate immunity. Nanotechnology, particularly smart multifunctional nanoplatforms that collectively work in targeted detection, signal amplification, controlled cargo release, and immune modulations are promising possibility to link diagnostics and therapy (Nooreldeen and Bach, 2021; Dong et al., 2023; Rother et al., 2024; Luo G. et al., 2025). Recent advancement in smart nanocarrier-based technologies considerably augmented the early detection and immune modulation strategies in lung cancer. Smart polymeric nanoparticles, especially PEGylated PLGA systems, showed great potential for targeted immune modulation by enhancing antigen presentation and reducing tumor-associated immune suppression (Zhang et al., 2022; Kesharwani et al., 2025). Similarly, lipid-based nanocarriers engineered with mannose or hyaluronic acid, such as hyaluronic acid-based polymer nanoparticles for tailored cancer therapy modifications, are capable of selectively targeting dendritic cells and tumor-associated macrophages, thus allowing for precise reprogramming of the tumor immune microenvironment (Paurević et al., 2024; Raval and Bhattacharya, 2025).

The immunotherapy, particularly immune checkpoint inhibitors (ICIs) targeting pathways such as PD-1/PD-L1 and CTLA-4, has become advanced in lung cancer management as it improves T-cell activation and also restores anti-tumor immune responses (Hu X. et al., 2023). The ICIs have revealed many noteworthy survival benefits in subsets of patients that lead to their approval as first-line and second-line therapies in non-small cell lung cancer (Liao et al., 2022). However, the clinical response remains variable due to tumor immune escape mechanisms, limited T-cell infiltration, heterogeneous expression of immune biomarkers, and immunosuppressive tumor microenvironments (Wang J. et al., 2022). Therefore, understanding the biological principles of immune checkpoint regulation and recognizing strategies to improve ICI efficacy, such as improved biomarker identification, combination therapies, and advanced delivery systems, remains a critical direction in lung cancer research (Mc Neil and Lee, 2025).

Lung cancer is a deadly malignancy because most patients are diagnosed at late stages when treatment options are limited (Lin et al., 2023). Early detection of lung cancer not only improves treatment possibilities but also significantly increases survival rates by reducing morbidity (Dama et al., 2021; Li et al., 2024a; Mohamed et al., 2024). Biomarkers present in blood, including circulating microRNAs, methylated cell-free DNA, proteins, autoantibodies, and extracellular vesicles, are emerging because these are less invasive for the early detection of lung cancer (Dama et al., 2021; Dama et al., 2025; Ma et al., 2025). At the same time, immune evasion and dysregulation are considered among the major lung cancer progression. Modulation of the immune microenvironment through different therapies, such as cellular vaccines (DC, T-cell, NK), immune checkpoint inhibitors, and immune signatures, has promising clinical and preclinical results (Wang D.-R. et al., 2022; Beg et al., 2025). Recent studies showed that immune profiles and signatures can serve dual roles both as prognostic indicators as well as predictors of response to immunotherapy (Zhang et al., 2024a; Chen et al., 2025c). So, integrating early detection with immune modulation offers a powerful dual approach to treat cancer at its most responsive stage and prevent its progression (Hu F. et al., 2025).

Nanotechnology is reshaping cancer immunotherapy because nanomaterials can deliver antigens and adjuvants, reprogram suppressive tumor microenvironment (TME), and improve the safety and efficacy of immune checkpoint and vaccination strategies (Wang et al., 2025). The preclinical and early clinical studies showed that smart, stimuli-responsive carriers and surface-engineered vectors markedly enhance the tumor selectivity and therapeutic index (Nirmala et al., 2023).

Notably, the physicochemical properties of nanoplatforms, including particle size, shape, surface charge, and material composition, play critical roles in measuring how these systems interact with immune cells within the TME (Lee et al., 2023). Smaller nanoparticles can improve lymphatic drainage and enhance dendritic cell uptake that promotes more efficient antigen presentation, while positively charged or surface-modified materials can reprogram M2 tumor-associated macrophages toward a pro-inflammatory M1 phenotype (Wei et al., 2022; Zheng et al., 2023). In the same way, properly engineered nanomaterials can enhance CD8^+^ T-cell infiltration, stimulate dendritic cell maturation, and overcome immunosuppressive cytokine environments. Therefore, tailoring nanoplatform design according to these physicochemical features is essential for achieving successful immune activation and reversing tumor-driven immune tolerance. Despite these advances, clinical translation faces challenges including manufacturing, long-term stability, and gaps between animal models and human responses. Addressing these barriers is important to understand the clinical potential of cancer nanomedicine (Wang and Zhang, 2023).

This review aimed to focus on the advancements in smart nanoplatforms designed for the early detection and immune modulation of lung cancer. It provides an integrated overview of recent progress in nanotechnology-based diagnostic tools, biosensors, and immunotherapeutic delivery systems that enhance sensitivity, targeting, and therapeutic efficacy. It also discusses design strategies, mechanisms of immune regulation, and current translational challenges in lung cancer. By highlighting innovative approaches and future directions, this work seeks to identify how nanotechnology can bridge the gap between early diagnosis and effective immunotherapy for the treatment of lung cancer.

## 2 Biological and molecular basis of lung cancer

Lung cancer is not only due to malignant epithelial cells but by a complex and dynamic ecosystem of stromal, vascular, and immune components. All these systems collectively define disease progression, therapeutic response, and metastatic potential (Chandra et al., 2025; Edirisinghe et al., 2025). TME of lung cancer is characterized by hypoxia, aberrant vasculature, extracellular matrix remodeling, and an inflammatory situation that stimulates proliferation and resistance to therapy. These nonmalignant parts actively communicate with cancer cells via cytokines, growth factors, extracellular vesicles, and metabolic crosstalk that create spatial and temporal heterogeneity, which complicates the diagnosis and treatment process. Understanding these types of interactions is essential when designing nanoplatforms that must indicate, sense, or reprogram the lung TME (Edirisinghe et al., 2025).

In lung cancer, hypoxic zones and abnormal vasculature decrease the oxygen and nutrient delivery, selecting for aggressive clones and impeding drug and nanoparticle penetration. Cancer associated fibroblasts and extracellular matrix hardening increase interstitial pressure and form physical barriers to delivery (Ciepła and Smolarczyk, 2024). Together, these features produce gradients of pH, redox potential, and enzyme activity that smart nanoplatforms can exploit for triggered release or targeted imaging. The lung’s unique architecture (large surface area, dual blood supply, and alveolar immune surveillance) further shapes both local tumor evolution and nanoparticle biodistribution, requiring lung-adapted delivery strategies (Arandhara et al., 2025).

### 2.1 Molecular biomarkers for early diagnosis and immune evasion mechanisms

Early detection relies increasingly on minimally invasive molecular biomarkers, which are detectable in blood, urine, or bronchoalveolar lavage. The main classes include circulating tumor DNA (ctDNA), circulating tumor cells (CTCs), tumor-derived extracellular vesicles/exosomes, and tumor-associated proteins and microRNAs. Advances in ultra-sensitive sequencing, PCR-based assays, and nanomaterial-enhanced biosensors have improved the limit of detection (LOD) for low burden disease (Kim and Park, 2023; Harisha et al., 2025). The combined biomarker, such as ctDNA alongwith protein or extracellular vesicle (EV) signatures, shows greater sensitivity and specificity than single analytes. Recent studies reported that ctDNA assays, especially when paired with other markers or imaging, hold the most promise for early stage lung cancer screening and minimal residual disease surveillance, though sensitivity in stage I disease remains a challenge. Smart nanodiagnostic platforms can amplify these weak signals through molecular capture, signal transduction, and multiplexed readouts (Lam et al., 2024; Ma et al., 2024; Li et al., 2025).

Lung tumors employ multiple overlapping immune escape strategies. These strategies include upregulation of immune checkpoints (PD-L1/PD-1), polarization of immunosuppressive myeloid populations (tumour-associated macrophages, myeloid-derived suppressor cells), secretion of suppressive cytokines (TGF-β, IL-10), metabolic competition (tryptophan depletion via IDO, lactate accumulation), and shedding of PD-L1 on exosomes (Jeong et al., 2025). These mechanisms blunt cytotoxic T-cell function and reduce antigen presentation, which limits the responses to immunotherapy in many patients. Targeting or reprogramming suppressive elements, such as repolarizing tumor associated macrophages (TAMs), blocking exosomal PD-L1, or locally delivering checkpoint inhibitors with nanoparticles, represents a rational route to restore antitumor immunity while minimizing systemic toxicity (Lin X. et al., 2024; Lv et al., 2025). The summary of main biomarkers, nanoplatform designs, and diagnostic/therapeutic strategies for lung cancer are given in Table 1.

**TABLE 1:** Summary of key biomarkers, nanoplatform designs, and diagnostic/therapeutic strategies for lung cancer.

Biomarkers for early detection of lung cancer					
Biomarker	Source	Clinical relevance (stage/feature)	Detection platforms	Comments	References
ctDNA (driver mutations)	Plasma	Early mutation detection, (minimal residual disease) MRD	NGS, nanoparticle-based enrichment	LOD is improved with bead capture	Xie et al. (2023)
Exosomal miR-21	Serum/plasma	Diagnostic/prognostic	Magnetic nanoparticle isolation, electrochemical sensor	Often enriched vs. total miRNA	Gao et al. (2021)
CEA (protein)	Serum	Common lung tumour marker	Gold nanoparticle immunosensor (colorimetric/SPR)	Limited specificity	Zhang et al. (2025b)
Circulating tumor cells (CTCs)	Blood	Metastatic potential	Microfluidic chip, antibody-functionalized NPs	Low abundance and enrichment is required	Cai et al. (2023)

Nanoplatform designs and immune-modulatory applications					
Nanoplatform	Core material	Immune target	Functional biomolecules	Advantage	​
Lipid nanoparticle (LNP)	Lipid bilayer	Antigen delivery for T cell priming	Tumour neoantigen mRNA, adjuvant	Clinical precedence (mRNA LNPs)	(Kiaie et al., 2022)
Iron oxide NP	Fe_3_O_4_	TAM repolarization, imaging	Toll-like receptor (TLR) agonist or small molecule repolarizer	Magnetic resonance imaging (MRI) visible; magnetic targeting possible	Wang et al. (2024d)
PLGA NP (polymeric)	PLGA	Local checkpoint blockade	Anti-PD-L1 siRNA	Controlled release; biodegradable	Deng et al. (2025)
Biomimetic exosome-mimic	Cell membrane-coated	Immune evasion neutralization	PD-L1 decoy peptides	Low immunogenicity; homotypic targeting	Srivastava et al. (2018)

Nanodiagnostic platform comparison					
Platform	Target analyte	Sensitivity	Throughput	Clinical readiness	​
Plasmonic nanoparticle sensor	Proteins/EVs	pg/mL – ng/mL	Low – medium	Preclinical	Kiio et al. (2024)
Nanostructured electrochemical sensor	ctDNA/miRNA	aM – fM	Medium	Preclinical/early translational	Gao et al. (2021)
Microfluidic-NP CTC chip	CTCs	1–10 cells/mL	Medium	Preclinical/early clinical	Huang et al. (2014)

## 3 Overview of nanotechnology in cancer research

Nanotechnology has improved oncology by enabling precise delivery, better diagnostics, and active modulation of the TME. Smart nanosystems engineered to sense local cues and respond with controlled release or signal generation are now central to efforts that push cancer care from empirical to precision paradigms. These multifunctional constructs combine targeting, imaging, and therapy to close the gap between early detection and effective, immune-aware treatment (Sun et al., 2023).

The evolution of nanomedicine spans simple drug loaded liposomes and polymeric carriers to advanced, stimulus responsive nanoplatforms and biomimetic systems. Early studies focused on improving pharmacokinetics and passive accumulation. The recent studies added active targeting (ligands, antibodies), controlled/triggered release (pH, enzymes, and redox), imaging labels, and immune modulatory cargos. Recently, lung cancer-focused work has emphasized route-specific delivery (including inhalable formulations), immune combination strategies, and modular platforms that permit rapid payload swapping for diagnostic or therapeutic functions (Alexandru et al., 2024).

### 3.1 Design principles of smart nanoplatforms and classification of nanomaterials used in lung cancer

**FIGURE 1:**
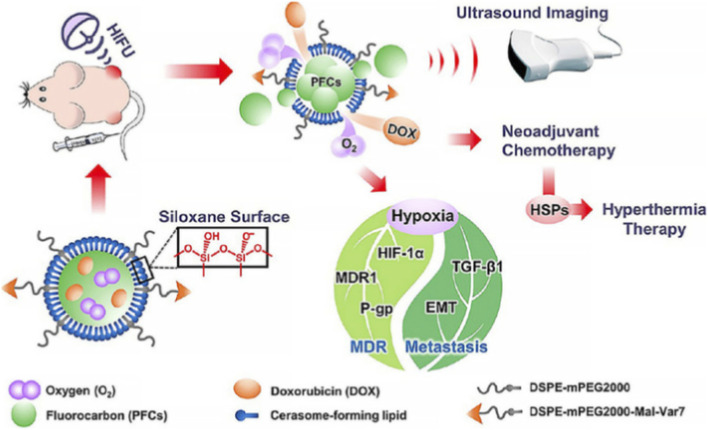
Ultrasound responsive nanodroplets are designed, fabricated, and capable of inhibiting tumor metastasis in the lung (Lin et al., 2022).

Smart nanoplatforms for the cancer diagnostics and therapy are engineered from interchangeable modules (core, payload, targeting ligand, and biomimetic shell) to allow tailored pharmacokinetics and multi functionality (Sun et al., 2023). TME cues pH, redox state, enzymes, and hypoxia are commonly exploited to trigger on-site drug release or immune stimulating activity, improving specificity and reducing off-target effects (Zhou W. et al., 2022). Stimuli-responsive polymers and nanogels enable controlled release kinetics and cargo protection (small molecules, nucleic acids, or adjuvants), supporting both early detection and immune modulation roles (Chang et al., 2021). Biomimetic strategies (cell membrane coatings and exosomes) enhance circulation time and immune compatibility while enabling antigen presentation or immune cell targeting for vaccination or reprogramming (Hu T. et al., 2023). Finally, modern designs emphasize TME remodeling and integrated theranostics to monitor response in real time, key for translation to lung cancer immunotherapy (Lu Q. et al., 2024). A stimulus-responsive drug delivery platform for the diagnosis and therapy of lung cancer is shown in Figure 1. In which oxygen and doxorubicin were loaded on nanodroplets, and high-intensity focused ultrasound (HIFU) was employed to trigger their controlled release while simultaneously enhancing ultrasound imaging for image-guided drug delivery. The application of mild temperature HIFU slightly increased the tumor temperature and improved local blood flow. As a result, ultrasound-induced oxygen release combined with moderate thermal elevation effectively alleviated tumor hypoxia and multidrug resistance. These synergistic effects significantly enhanced the therapeutic efficacy of doxorubicin against lung metastases (Lin et al., 2022). The nanomaterials used for lung cancer can be grouped into four major classes, each with distinct strengths and limitations for detection and immune modulation. A concise classification and representative examples of nanomaterials used for lung cancer are given in Table 2.

**TABLE 2:** Classification of nanomaterials used in lung cancer.

Class	Representative examples	Primary functions in lung cancer	Advantages	References
Organic	Liposomes, lipid nanoparticles, polymeric NPs (PLGA, PEGylated polymers)	Drug/siRNA delivery, inhalable formulations, vaccine delivery	Biodegradable, clinically validated platforms; easy payload loading	Kapoor, et al. (2025)
Inorganic	Gold, iron oxide, silica, quantum dots	Imaging contrast, photothermal/photodynamic therapy, and immune adjuvants	Strong physical properties (optical, magnetic); long persistence requires surface engineering	Wang et al. (2022c)
Hybrid	Lipid-inorganic, polymer-inorganic composites, mesoporous silica with polymer shells	Combined imaging, controlled release, targeting	Tunable multifunctionality; balance between stability and biodegradability	Chan et al. (2022)
Biomimetic	Cell membrane-coated NPs, exosome-mimetics	Immune-evasive delivery, antigen presentation, enhanced uptake by immune cells	Low immunogenicity, improved circulation and cell targeting; promising for immune modulation	Han et al. (2024)

## 4 Early detection in lung cancer by smart nanoplatforms

**FIGURE 2:**
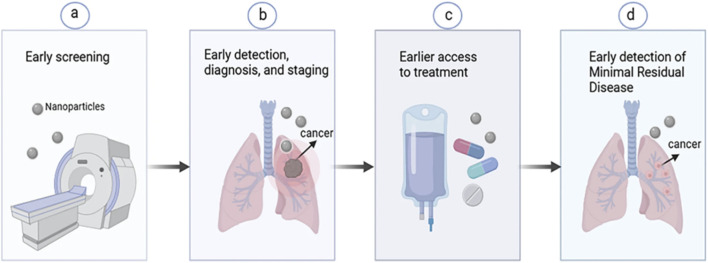
Role of nanotechnology at all stages of lung cancer diagnosis and treatment. **(a)** Early screening of lung cancer, **(b)** Early detection, diagnosis, and staging, **(c)** Early access to treatment to promote lung cancer therapy, **(d)** Nanotechnology promotes the detection of MRD in lung cancer (Feng J. et al., 2024).

Early detection of lung cancer remains the single most important factor for survival. Smart nanoplatforms combine with nanoscale sensing materials, targeted contrast agents, miniaturized devices, and artificial intelligence (AI) driven data fusion to increase sensitivity and specificity. These features inhance the efficiency of biomarkers, circulating DNA and RNA, exosomes, proteins, and imaging signatures. Nanoplatforms aim to move diagnosis earlier in the disease timeline, as shown in Figure 2, by enabling low-volume sampling of blood, breath, saliva, point of care (POC) workflows, and multi modal data integration that can uncover subtle disease signals that are not possible by conventional assays (Chaudhary et al., 2024; Mikaeeli Kangarshahi et al., 2024; Bartolomucci et al., 2025).

**FIGURE 3:**
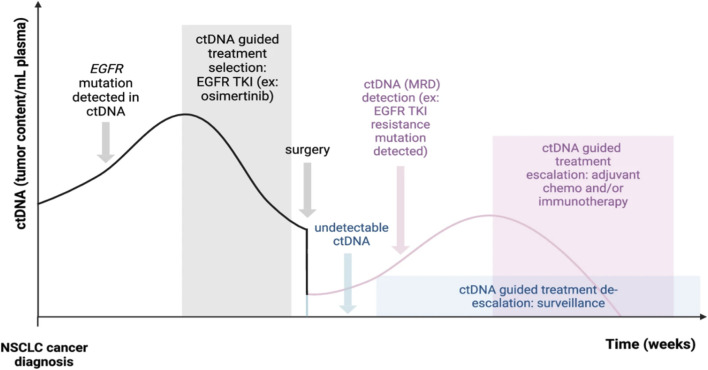
Current ctDNA precision oncology applications in Non-Small Cell Lung Cancer (NSCLC) (Bartolomucci et al., 2025).

Nanomaterial enabled biosensors, including electrochemical, optical, and chemiresistive types, have significantly enhanced detection sensitivity for circulating tumor biomarkers in lung cancer. New approaches, including nucleic acid hybridization sensors, nanoparticle augmented fluorescence assays, and plasmonic or exosome capture platforms, allow for precise identification of ctDNA, microRNA, and tumor derived exosomes at attomolar (10^−18^ M) to picomolar (10^−12^ M). These approaches required low sample volumes to support early molecular diagnosis and real time monitoring of disease, as shown in Figure 3 (Yang et al., 2024; Bartolomucci et al., 2025). Integration of selective surface chemistries, aptamers, peptide and protein capture ligands with signal-amplifying nanostructures like gold nanoparticles, carbon nanotubes, and graphene has produced model assays. These assays approach clinical sensitivity while reducing times compared with standard PCR or sequencing-based workflows (Kim et al., 2024; Yin et al., 2024). Furthermore, challenges remain in standardization, matrix effects from complex biofluids, and validating clinical utility in large cohorts (Yin, et al., 2024; Bartolomucci et al., 2025). A summary of recent nanoplatforms for the early detection of lung cancer is given in Table 3.

**TABLE 3:** Summary of recent nanoplatforms for the early detection of lung cancer.

Nanoplatform	Biomarker	Biological target	Sensitivity	References
High-throughput Nano-biochip Integrated System for Liquid Biopsy	EV membrane proteins (CD81, PDL1, GLIPR1, LBR and SFTPA1)	Plasma extracellular vesicle	AUC: 0.931 Sensitivity; 89.4%	Han, et al. (2025)
AI-assisted SERS profiling of plasma exosomes on plasmonic substrates	Exosome molecular	Plasma exosomes	AUC; 0.84, sensitivity; 83.3%	Lu et al. (2024a)
Multi-receptor SERS sensor (BC/AuNP film)	Exosome surface binding signatures	Plasma exosomes	Sensitivity; 90%	Lu et al. (2024b)
Electronic-nose; eNose (chemiresistive nanostructured sensor array)	VOC pattern signature (breath)	Exhaled breath	AUC; 0.89 Sensitivity; 0.90	Lee et al. (2024)
Prospective eNose	VOC breath signature	Exhaled breath	---	de Vries et al. (2023)
Nanomaterial-assisted electrochemical platform (CM-OECATs nanocomposite)	Multiplex protein panel	Serum clinical samples	AUC; 0.9748	Wan et al. (2024)
CRISPR-Cas12a enhanced electrochemical ctDNA sensor using MB/Fe_3_O_4_@COF/PdAu	EGFR L858R/EGFR activating mutations (ctDNA)	Plasma cfDNA/ctDNA	---	Liu et al. (2022)
Exosomal miRNA panel (serum exosomes) using sequencing, qRT-PCR validation	Combined 3-miRNA panel (miR-200b-3p, miR-3124-5p, miR-92b-5p)	Serum exosomes	AUC; 0.93	Kim et al. (2023)

surface-enhanced Raman spectroscopy (SERS), gold nanoparticles onto bacterial cellulose (BC/Au NPs, film), CNT-doped MXene, incorporated into an organic electrochemical transistor aptamer sensor (CM-OECATs).

Nanotechnology provide targeted delivery for nearly every major medical imaging modality, enabling both improved detection sensitivity and molecular specificity in lung lesions. For example, iron oxide and gadolinium bearing nanostructures that enhance MRI signal in tumor microenvironments, radiolabeled nanoparticles for positron emission tomography (PET) that increase tumor to background contrast, and plasmonic or dye loaded nano systems for optical and photoacoustic imaging that allow high-resolution mapping of superficial or surgical specimens (Feng X. et al., 2024; Chow, 2025; Cai et al., 2024). Theragnostic designs combine imaging and therapy, such as, photothermal or radio-sensitizing nanoparticles, supporting see and treat paradigms. However, their clinical translation requires rigorous biodistribution, toxicity, and regulatory evaluations because nanoparticle behavior in lung tissue and circulation can be complex (Hazra et al., 2022; Haghayegh et al., 2024).

### 4.1 Point of care and wearable nano diagnostic technologies and artificial intelligence in nano diagnostics

POC wearable nano-diagnostic devices and POC biomedical sensors are transforming decentralized and rapid lung cancer screening by facilitating sensitive and real-time testing at the bedside. New nanotechnology breath-based chemiresistive sensor arrays and nose on chip systems employing functionalized nanomaterials can reliably identify volatile organic compounds (VOC) identifies unique to lung tumors, as a highly specific, noninvasive diagnosis (Chaudhary et al., 2024). While paper or microfluidic-based nanoparticle-assisted lateral flow and smartphone read electrochemical tests enable near-patient ctDNA/protein readouts and Cancer Antigen, like CA-125 (Pourmadadi et al., 2023; Yang et al., 2024). Wearable patches and flexible sensors constructed from bioinspired nanomaterials offer continuous physiological and biomarker monitoring, potentially identifying temporal variations and early signs of malignancy. Ensuring robustness, minimizing user variability, and maintaining data accuracy remain active research areas that must be addressed before these technologies achieve reliable and widespread clinical application (Khalifa and Albadawy, 2024).

Data integration and Artificial Intelligence (AI) have greatly improved the diagnosis of cancer. AI is revolutionizing how nano diagnostic results are interpreted and merged with clinical data. Machine learning algorithms enhance signal amplification from noisy outputs of nano-sensors, assist in multi-analyte signature classification, such as integrating ctDNA fragment patterns and VOC panels, and allow multimodal imaging, biomarker, and patient metadata fusion for enhancing diagnostic accuracy and risk stratification (Carrillo-Perez et al., 2022; Chaddad et al., 2023). These centers, such as multimodal data fusion, integrate with imaging, biomarker, and patient metadata to enhance diagnostic accuracy, risk assessment, and individualized treatment planning. In addition, AI technologies are speeding up nanoparticle design by forecasting ideal physicochemical features, optimizing targeting performance, and calibrating assay parameters for more efficient and reproducible performance (Alavinejad et al., 2025). Currently, collaborative methods are under development to enable collaborative model training across institutions while not violating the privacy of patient data, and explainable AI models seek to enhance transparency and clinical trust. Despite the various challenges that still exist, such as dataset heterogeneity, possible algorithmic bias, restricted access to large annotated clinical datasets, and the absence of standardized regulatory mechanisms for validation and approval of AI-fused diagnostic tools (Zhu et al., 2025). Overcoming these challenges is a requirement to bring AI-boosted nano diagnostics into stable, ethical, and clinically viable forms that can enable early cancer diagnosis and precision medicine.

## 5 Nanotechnology driven immune modulation strategies

Recent developments in nanotechnology have transformed immune modulation strategies to boost anti-tumor immunity by targeted and controlled immune activation. Current research demonstrates that theranostic nanoparticles can simultaneously deliver diagnostic imaging and immunotherapeutic benefits and induce strong and focal immune stimulation in models of lung cancer (Muradova et al., 2025). These nanoplatforms are multifunctional in their ability to monitor therapeutic response in real time while activating immune effector cells within the tumor microenvironment. Inhalable drug delivery systems containing IL-12 mRNA in exosomes or nanobubbles have been very promising as it enables localized delivery to lung tissue, which leads to strong activation of the immune system with significantly reduced systemic toxicity and inflammatory side effects (Liu et al., 2024). In addition, nanomedicine platforms that utilize next-generation antigen based platforms are being engineered to improve the presentation of antigens, facilitate co-delivery of co-stimulatory adjuvants, and induce maturation of dendritic cells, important processes for initiating efficacious and personalized cancer immunotherapy (Lin L. et al., 2024). New advances in nano immunotherapy have also pointed towards opportunities for leveraging stimuli responsive processes, including pH or enzyme activated release, as well as immune checkpoint inhibitor co-delivery and tumor microenvironment modulation to help circumvent resistance to therapy (Wang et al., 2025). Recently, inhalational nanocarriers have received major attention because they bypass hepatic clearance and deliver immunomodulatory agents directly into lung tissues, thereby achieving much higher therapeutic concentrations with reduced systemic toxicities. The inhalable liposomal formulations of PD-L1 siRNA or STING agonists have shown potent immune activation in preclinical lung cancer models (Liu et al., 2019; Ge et al., 2025). Smart inhalable metal-organic framework-based carriers functionalized with pH-responsive gates that can co-deliver chemotherapeutics and immune stimulants with real-time release control have shown promising outcomes in tumor regression and immune reprogramming (Chen et al., 2025d; Yan et al., 2025). Additionally, these smart nanotechnologies-mediated immune strategies made a significant advancement toward targeted, individualized, and effective lung cancer immunotherapy.

### 5.1 Immune checkpoint targeting using nanocarriers and nanovaccines and antigen delivery systems

Nanocarriers like lipid nanoparticles, polymeric particles, protein-based carriers, and hybrid nanosystems are under active development to improve targeted delivery of immune checkpoint inhibitors like anti-PD-1, PD-L1, and CTLA-4 antibodies, siRNA, and mRNA constructs directly to the TME (Guo L. et al., 2025). Targeted delivery significantly increases therapeutic efficacy while reducing systemic toxicity and immune related adverse events typically encountered with traditional checkpoint blockade treatments. By encapsulating these checkpoint blocking agents, nanocarriers maintain protection against enzymatic degradation, improve localized drug accumulation in tumors, and allow for controlled or stimulus sensitive release mechanisms induced by conditions like pH, enzymatic activity, or exposure to light (Hu J. et al., 2025). This control of space-time delivers sustained therapeutic concentrations and also targeted immune modulation within the TME. Furthermore, co-delivery strategies where checkpoint inhibitors are mixed with immunostimulatory cytokines, adjuvants, or tumor antigens in a single nanoplatform have reported synergistic enhancement of innate and also adaptive immunity, leading to enhanced rates of response in cancer models preclinically (Tian et al., 2024). While these encouraging results hold much promise, various challenges must yet be overcome before clinical translation can be successful, such as large-scale and reproducible production of nanoparticles, long-term stability upon storage and in circulation, possible immunogenicity, and the development of standardized test protocols. Ongoing formulation design optimization as well as regulatory validation will be critical to achieve the full clinical value of nanocarrier based immune checkpoint delivery systems.

Nanovaccines take advantage of advanced carrier systems such as liposomes, polymeric nanoparticles, virus-like particles, and self-assembling protein constructs to co-deliver tumor antigens in association with immunostimulatory adjuvants to improve antigen presentation and elicit potent T-cell activation (Zhang et al., 2019). These nanocarriers ensure the efficient engulfment by antigen presenting cells (APCs) and provide sustained, controlled antigenic material release, ensuring extended immune stimulation. Current advances in nanotechnology have provided new vaccine platforms, such as lipid nanoparticle (LNP)-based mRNA vaccines, peptide polymer conjugates, and dendritic cell-targeted nanoparticles, enabling targeted delivery to lymph nodes and effective induction of cytotoxic T-lymphocyte responses for powerful antitumor immunity (Tian et al., 2024). Moreover, cutting-edge studies identify the modular and tunable nanovaccine architectures for use in personal neoantigen immunotherapy to provide scalable and tunable manufacturing to cover unique tumor patterns. These developments altogether are a significant step ahead towards the next-generation of cancer vaccination approaches (Zhang et al., 2019; Saleh et al., 2025).

### 5.2 Modulation of tumor associated macrophages and T-cell activation

**FIGURE 4:**
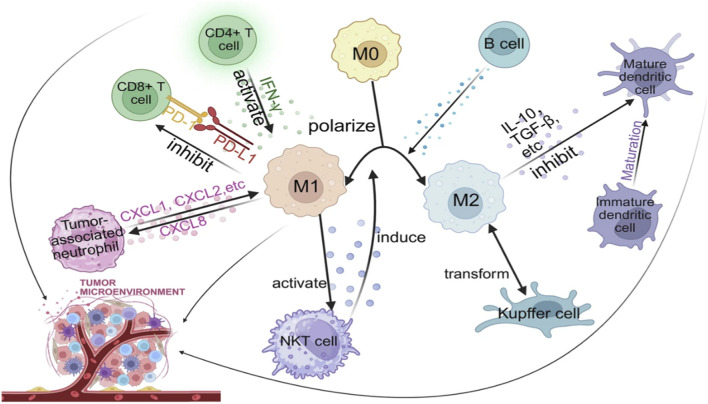
Schematic diagram of tumor-associated macrophage (TAM) interactions in the tumor microenvironment, illustrating M1/M2 polarization and communication with immune cells (T cells, DCs, TANs, and B cells) that control antitumor immunity and tumor growth (Xu et al., 2025).

TAMs are among the most common immune cells of the TME and are a major regulator of immunosuppression, tumor growth, angiogenesis, and metastasis (Li et al., 2024a). TAMs have a tendency to acquire an M2 like phenotype that supports tumor cell proliferation, suppresses cytotoxic T-cell functions, and facilitates immune evasion. As a result, TAMs have emerged as a valuable target for cancer immunotherapy. Nanotechnology brings novel and targeted ways to control TAM activity by using three broad strategies: (i) blocking circulating monocyte recruitment that gets differentiated into TAMs, (ii) targeted elimination of the pro-tumoral M2 subset macrophages, and (iii) reprogramming the M2 macrophages into anti-tumoral M1 phenotypes with the ability to secrete pro-inflammatory cytokines and restore antitumor immunity (Kim et al., 2024). Macroscopic effects to be brought about by them have led to the development of macrophage receptor ligand-functionalized nanocarriers that deliver therapeutic molecules like CSF-1R inhibitors, siRNA, or TLR agonists to TAMs selectively and thereby modulate the immune system effectively (Li et al., 2024b; Xu et al., 2025). n recent times, lipid nanoparticles (LNPs) loaded with RNA have also been identified as a highly promising delivery strategy for *in vivo* reprogramming of macrophages due to their biocompatibility, efficient cell uptake, and ability to protect against RNA cargo degradation [30]. The LNPs have the properties to efficiently modulate gene expression in macrophages, changing TME from immunosuppressive to immunostimulatory states. Additionally, nanoparticle platforms can co-deliver cytokines or co-stimulatory molecules to trigger T-cells simultaneously, thus integrating innate and adaptive immunity. Such combinational macrophage–T-cell targeting approaches bear significant potential for the next-generation of immuno-oncology treatments, as shown in Figure 4 (Gül et al., 2025; Xu et al., 2025).

Nanoplatforms are areas of research that offer scope to combine various therapeutic modalities chemotherapy, radiotherapy, photothermal therapy (PTT), photodynamic therapy (PDT), and immunotherapy, on a single scaffold (Naik et al., 2025). These multifunctional systems allow for concurrent tumor killing and immune stimulation. For instance, drug carrier nanoparticles or photosensitizer-loaded nanoparticles can induce tumor cell death as well as induce *in situ* release of tumor-associated antigens. When used in combination with immune checkpoint blockade, the process augments systemic antitumor immunity, creating an *in situ* vaccine effect that enhances the body’s immune response against metastatic or remaining cancer cells (Sun et al., 2024; Naik et al., 2025). Additionally, photothermal and radiotherapy derived nanoplatforms have shown striking synergistic effects by recording more effective tumor regression and greater abscopal responses when combined with immune adjuvants or checkpoint inhibitors. Rational and spatiotemporal design of these multimodal nanotherapies is fundamental to providing clinical efficacy, reducing toxicity, and maximizing therapeutic safety and translation toward next-generation cancer treatment strategies (Pan et al., 2024; Sun et al., 2024; Naik et al., 2025).

## 6 Design features of smart nanoplatforms

Nanotechnological intelligent nanoplatforms are highly developed to encompass stimulus-responsiveness and active-targeting properties that respond specifically to tumor microenvironmental signals including acidic pH, redox gradients, increased reactive oxygen species (ROS), and tumor-associated enzymes (Sun et al., 2023; Sabit et al., 2025). Such systems facilitate drug release under control, enhanced tumor penetration, as well as decreased off-target toxicity by triggering only in pathological conditions. In addition, to increase tumor selectivity, nanocarriers are targeted with ligands such as antibodies, peptides, aptamers, and small molecules that enable receptor-mediated uptake and enhance accumulation at the tumor site (Urmi et al., 2024; Omidian et al., 2025). Based on recent reports, hybrid nanotherapeutic strategies combining several stimuli responsive mechanisms including combinations of redox and pH sensitivity, or enzyme and ROS responsiveness with ligand based targeting have proved synergistic gains in therapeutic index and tumor selectivity (Parra-Nieto et al., 2024). These multiscale systems adaptively interact with the heterogeneous tumor microenvironment, facilitating site-specific and controlled drug release to reduce off-target toxicity. Further, the multivalency, spatial orientation, and density of immobilized ligands significantly contribute to regulating cellular binding efficiency and receptor-mediated uptake, directly impacting therapeutic efficacy. Dual targeting strategies, which selectively bind to both tumor-specific as well as immune-related surface markers, are being introduced as sophisticated next-generation tools for precision oncology (Navaneeth and Karthikeyan, 2024; Omidian et al., 2025). Concurrently, the evolution of computational modeling, molecular dynamics simulations, and rational nanomaterial design over the last few years has enabled the development of tumor-microenvironment-adaptive carriers with pharmacokinetic optimization, enhanced cellular internalization, extended systemic circulation time, and improved *in vivo* stability, thus guaranteeing better therapeutic outcomes (Hou et al., 2025; Sabit et al., 2025).

### 6.1 Multifunctional and biocompatible theranostic nanoplatforms

**FIGURE 5:**
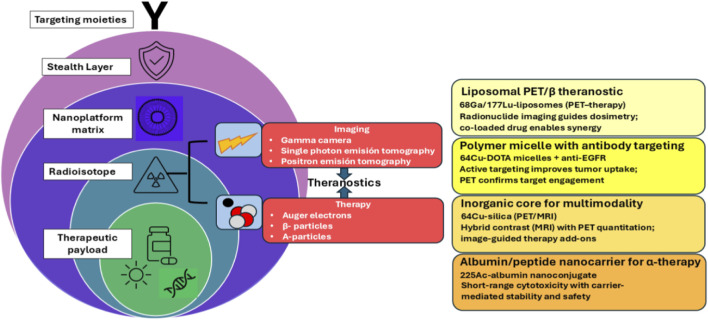
Modular theranostic nanoplatforms in nuclear medicine (Salgueiro and Zubillaga, 2025).

Nanomedicine focuses on multifunctional theranostic nanoplatforms that both possess therapeutic and diagnostic functions and are highly biocompatible and pharmacokinetically predictable. These smart nanoplatforms deliver drugs, genes, or immunomodulators in combination with imaging modalities, including MRI, PET, fluorescence, or ultrasound, enabling real-time monitoring of biodistribution, therapeutic efficacy, and early therapeutic response expressed in Figure 5 (Yasir et al., 2024; Salgueiro and Zubillaga, 2025).

Further hybrid inorganic-organic and polymeric architectures combine photothermal or photodynamic modules with targeting ligands and contrast agents, enabling precision-guided therapy and simultaneous visualization at the molecular level (Salgueiro and Zubillaga, 2025). Moreover, the clinical success of such advanced systems depends on safe material composition and controlled *in vivo* behavior. Important physicochemical characteristics, particle size, charge, hydrophilicity, and degradability dictate circulation half-life, reticuloendothelial system clearance, and tissue uptake (Kyriakides et al., 2021; Saker et al., 2024). Rational design, therefore, requires the careful balance of multifunctionality with biological safety. Emerging methods utilize biodegradable polymers and environmentally friendly nanomaterials to minimize chronic toxicity and immune stimulation without sacrificing efficacy (Kyriakides et al., 2021; Parra-Nieto et al., 2024). In the future, pharmacokinetic and immunotoxicity assessment frameworks are necessary to provide reproducibility and regulatory acceptability and make them scalable and safe theranostic nanomedicine (Saker et al., 2024; Sabit et al., 2025).

## 7 Translational barriers and challenges

While promising progress has been achieved in nanotechnology-based cancer therapeutics, it is difficult to translate these systems into efficient lung cancer treatments. Biological barriers like mucus, surfactant, and heterogeneous tumor vasculature restrict the penetration and retention of nanoparticles in lung tissue. The complexity of the tumor microenvironment, i.e., hypoxia and stromal desmoplasia, also impedes accumulation of targeted drugs (Tong et al., 2024). Scale up and reproducible production of nanocarriers is hampered by technical challenges related to stability, batch homogeneity, and manufacturing practice compliance. Routine uncertainty, in the form of inconsistent evaluation paradigms between the Food and Drug Administration (FDA) and European Medicines Agency (EMA), persists to hinder the clinic transition of cancer nanomedicines (Shi et al., 2017). Further, expensive production and characterization complexity disallow scalability and accessibility. Conversely, long-term biosafety concerns such as nanoparticle deposition, immune stimulation, and unpredictable off-target toxicity, pose strong translational risks (Zhang et al., 2024b). To fill these gaps, future strategies focus on patient-tailored nanoformulation design, AI-driven toxicity prediction, and harmonized regulatory frameworks for clinically safe translation (Zhang et al., 2023).

### 7.1 Manufacturing and biological barriers in lung targeted nanomedicine

Selective and targeted delivery of intelligent nanomedicines to the lung is associated directly with anatomical and biological barriers that restrict therapeutic effectiveness. The respiratory tract is shielded by a cascade of defense barriers, such as mucus, pulmonary surfactants, and epithelial cell barriers, which all retard nanoparticle adhesion, deposition, and penetration in target tissue (Cojocaru et al., 2024). Nanoparticles must counteract mucociliary clearance mechanisms that rapidly remove foreign particles from airways, as well as evade phagocytic uptake by alveolar macrophages that can greatly cut short their sojourn time and reduce drug bioavailability in the target site (Fernández-García and Fraguas-Sánchez, 2024; Wang et al., 2024c). In addition, pathological alterations like inflammation, fibrosis, and tumor-induced airway architecture remodeling change permeability of the tissue and establish heterogeneous microenvironments that impede nanoparticle diffusion and uniform distribution in sites of tumors (Deng et al., 2021; Sanati et al., 2025). All these barriers individually highlight the need to develop lung-specific nanocarriers with well-designed aerodynamic properties, surface modifications, and targeting strategies to maximize pulmonary delivery and therapeutic effects.

Recent studies have shown that surface engineering, such as PEGylation or charge modulation, improves mucosal diffusion and minimizes immune clearance (Deng et al., 2021; Fernández-García and Fraguas-Sánchez, 2024). Current advances in inhalable nanocarriers, such as lipid-based aerosols and polymeric micelles, show enhanced delivery efficiency and tumor penetration when aerodynamically optimized for size and surface properties (Jin et al., 2023; Chen R.-K. et al., 2025). Yet, with good preclinical performances, scaling up nanotherapeutics into large scale manufacturing continues to be an unprecedented challenge. Laboratory synthesis pathways, emulsification, nanoprecipitation, and microfluidics do not readily scale up to replicate consistent particle size, surface charge, and encapsulation efficiency (Operti et al., 2021). Continuous manufacturing and microfluidic-based platforms are becoming the solutions to obtain consistent particle batches and minimize process variability (Feng et al., 2019; Abdelmonem et al., 2025). In addition to that, ensuring their regulatory approval and batch-to-batch reproducibility demands sophisticated analytical characterization equipment and rigorous quality control, which considerably escalates production costs (Operti et al., 2022; Khopade and Shah, 2025).

### 7.2 Safety, ethical and regulatory, challenges in nanomedicine translation

Safety and biocompatibility of nanocarriers are the key issues for clinical translation. Biodegradable polymers, such as polyethylene glycol-poly(lactic-co-glycolic acid (PEG-PLGA), chitosan, and lipid-based nanocarriers, represent a widely used family of materials owing to their relatively low immunogenicity and favorable clearance profile (Wu et al., 2024). The PEGylation indeed reduces opsonization and prolongs the circulation time by minimizing off-target accumulation within the liver and spleen (Zhang et al., 2022; Kesharwani et al., 2025). Nevertheless, the main problem of nanoparticle accumulation in the reticuloendothelial system cannot be completely ignored. Long-term exposure to non-degradable nanoparticles results in oxidative stress, inflammation, and organ-specific toxicity. Recently reported surface modification strategies, including zwitterionic coating or HA-decoration, may markedly minimize the uptake of macrophages and systemic toxicity while maintaining therapeutic efficacy (Li et al., 2022; Skorzynski et al., 2025). Moreover, poorly soluble nanocarriers and inhalable nanocarriers targeting lung tissue are designed for maximal local deposition and minimal systemic exposure, offering further advances in improving the safety profile in preclinical models (Anderson et al., 2020; Feng X. et al., 2024). Therefore, safety is balanced against therapeutic benefit by rational design, optimization of drug dose, and careful monitoring of the biodistribution of nanoparticles.

Smart Nanomedicines also have different regulatory problems because of their physicochemical and biological dual hybrid nature. Agencies such as the FDA and EMA stress thorough physicochemical characterization, particle stability testing, and immunotoxicology profiling prior to approval (Soares et al., 2018; de Vlieger et al., 2019). Even harmonized international guidelines only slow down global harmonization and product approval. Ethical issues also come from the unresolved long-term biodistribution and possible unforeseen immune or genetic effects. Moreover, the high development and production costs are concerns regarding accessibility, especially in low- and middle-income economies. Open risk benefit evaluation, patient consent measures, and open data sharing are necessary to enhance clinical trust and provide an equal access approach to nano therapies (Wang L. et al., 2024; Khopade and Shah, 2025). Long-term safety is a gigantic concern while translating nanomedicine. Long-term accumulation of inorganic nanoparticles in organs like the liver, spleen, and kidneys is known to cause chronic inflammation, fibrosis, or oxidative stress. Research has shown that gold and silica nanoparticles cause mild but persistent tissue alterations months after they have been administered (Paranjpe and Müller-Goymann, 2014; Abdelmonem et al., 2025; Chen L et al., 2025). Moreover, nanoparticle interactions with the immune system can lead to complement activation, imbalance of cytokines, or even a change in microbiota composition (Soares et al., 2018; Garcés et al., 2021; Sanati et al., 2025). Per current research, biodegradable polymers like PLGA or lipid-based systems present with better clearance profiles, but stringent long-term monitoring must be maintained to establish metabolic safety. The integration of advanced *in vitro* organ-on-chip models and longitudinal *in vivo* imaging is increasingly proposed to predict and mitigate chronic nanotoxicity (Soares et al., 2018; de Vlieger et al., 2019).

## 8 Future perspectives and emerging directions

Lung cancer always remains a major partner or contributor to global cancer mortality, responsible for about one in five cancer deaths, even with notable improvements in early detection and treatment methods (Siegel et al., 2022; Bray et al., 2024). Now a days its treatment paradigm has evolved significantly, moving away from conventional chemotherapy toward precision medicine and immunotherapy, which focus on targeting the molecular and immunological profiles of tumors (Swanton and Govindan, 2016; Herbst et al., 2018). Nanomaterials have opened a number of promising avenues in the early detection and precision diagnostics of lung cancer. The so-called theranostic nanocarriers combine imaging and therapeutic capability in one multifunctional platform, enabling tumor visualization and targeted treatment in parallel (Baskaran et al., 2024). Further improvements in stimuli-responsive nanocarriers, including pH-sensitive and ROS responsive systems, have increased the potential for real-time monitoring of tumor microenvironments and provided dynamic tracking of therapeutic response *in vivo* (Zhang Y. et al., 2025). Integration of nanomaterials with liquid biopsy approaches such as exosome capture, and ctDNA sensing, has improved the sensitivity and specificity of non-invasive diagnostics of lung cancer (Tang et al., 2024; Wu et al., 2025). In the near perspective, the development of multimodal nano-sensors capable of detecting early molecular alterations coupled with wearable or implantable monitoring devices may result in a transformation of lung cancer management [6]. Finally, the ability to combine targeted delivery, real-time imaging, and biosensing within one nanoplatform enables personalized detection and therapy, thereby reducing morbidity and improving patient survival (Hua et al., 2025). Now a days, ICIs such as pembrolizumab, nivolumab, atezolizumab, and durvalumab have transformed the treatment of early and late stage NSCLC by achieving long lasting responses in subsets of patients (Reck et al., 2016).

Yet, primary and secondary resistance, tumor heterogeneity, and restricted biomarker predictability continue to pose dominant clinical hurdles. As a result, research today is moving in the direction of personalized neoantigen vaccines, multi-modal immunotherapy, precision medicine guided by biomarkers, and AI-driven analytics and nanomedicine platforms combined together for improved diagnosis, therapy optimization, and enhanced patient outcomes (Ott et al., 2017; Mellman et al., 2023).

### 8.1 Integration of nanotechnology with personalized medicine

**FIGURE 6:**
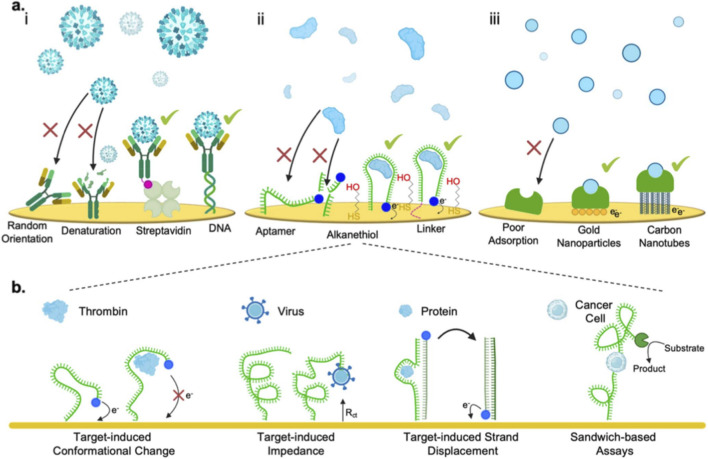
Common types of biorecognition elements used in biosensing and their respective immobilization strategies: **(a)** (i) Antibody-based systems. (ii) Nucleic acid-based systems. (iii) Molecularly imprinted polymer-based systems. **(b)** Various signal transduction strategies of aptasensors (Akhlaghi et al., 2024).

The combined system of personalized medicine and nanotechnology is an efficient step forward in precision medicine, which makes it possible for individual diagnosis, of multiple targeted treatments, and therapy outcome monitoring in real-time. Collectively, these nanocarriers can also be formulated with real-time or “intelligent” features in combination with imaging probes including near-infrared dyes, magnetic nanoparticles, and photoacoustic reporters for simultaneous therapy and monitoring. For instance, ROS-responsive polymeric nanocarriers encapsulating fluorescent reporters enable real-time tracing of drug release in tumor therapy (Zhang et al., 2021; Li X. et al., 2023). While magnetic-response nanoplatforms have been developed to deliver immune modulators and allow MRI-guided monitoring of treatment response (Yang et al., 2022; Wei et al., 2026). Overall, all these recent designs underline the importance of smart nanocarriers in establishing targeted, efficient, and responsive immunotherapy in lung cancer (Zhou L. et al., 2022; Feng J. et al., 2024). Nanotechnology is concerned with the manipulation and engineering of materials at the nanoscale (1–100 nm), a scale that is similar to biomolecules, so as to achieve very specific interaction with cellular and molecular targets (Salata, 2004; Wagner et al., 2006). In personalized medicine, different nanomaterials like liposomes, dendrimers, polymeric nanoparticles, metal nanoparticles, carbon nanotubes, and quantum dots are utilized for the delivery of drugs and genes as per the individual’s molecular profile (Mura et al., 2013; Zhou Y. et al., 2022). Such nano-systems are feasible to be engineered using ligands, antibodies, or aptamers in order to selectively target tumor-specific biomarkers and thus escape the redundant toxicity and enhance treatment specificity and overall efficacy (Ventola, 2017). Moreover, nanotechnology has revolutionized molecular characterization and biorecognition element design used in biosensing platforms. The novel immobilization techniques, nanoscale sequencing and biosensing devices enable the facilitation of accurate pharmacogenetic analysis using the identification of genetic polymorphisms and mutations that influence personalized therapy outcomes. These advances in technologies allow dynamic adjustment of treatment regimens and real-time patient stratification. In total, the interaction between personalized medicine and nanotechnology provides an unparalleled platform for delivering ultra-specific, effective, and adaptive personalized healthcare solutions. Such paradigmatic shift not only enhances diagnostic precision and therapeutic benefits but also enables the formation of predictive, preventive, and participatory medicine by filling the gap from molecular to clinical translation as depicted in Figure 6 (Jain, 2019; Akhlaghi et al., 2024).

The advanced innovations in nano-diagnostics and theranostics have also coupled therapy and imaging in one nanoplatform, permitting real-time monitoring of drug release and disease progress.

### 8.2 Role of AI, machine learning, and digital twins in smart nanomedicine

The use of nanomedicine is aggressively growing and will see tremendous growth in the healthcare industry. Nanomedicines involve a broad spectrum of formulations, such as liposomes, lipid nanoparticles (LNPs), antibody drug conjugates ADCs, polymeric nanoparticles, viral vectors, cell-derived nanoparticles, inorganic nanoparticles, nanocrystals, protein-based nanoparticles, and nano-micelles (Thapa and Kim, 2023). As a result, they have been extensively used to treat cancer, infectious diseases, and neurological disorders. But clinical applications of nanomedicines on a larger scale are hindered, mainly because of limitations such as poor in vitro-in vivo correlation, off-target-derived toxicity, intricate processes of manufacture, and product instability (Agrahari and Agrahari, 2018). The current advancements in AI and Machine Learning (ML) have provided promising solutions to overcome the current challenges in nanomedicine development. AI/ML tools can predict, analyze, and optimize complex systems based on large sets of data, thus aiding multiple steps in the design, production, and clinical testing of nanomedicine (Kolluri et al., 2022).

### 8.3 Lung cancer care through next-generation bioinspired smart nanoplatforms

Nanotechnology is the most revolutionary science of the 21st century, encompassing nanometer-scale engineering and production of materials (1–1000 nm), at which most biological processes naturally take place (Surendiran et al., 2009). Use of nanotechnology in medicine, known as nanomedicine, aims to create nanoscale therapeutic systems for better patient outcomes. Among them, nanoparticles (NPs), particularly those with a size of 10–100 nm, are highly useful owing to their capability to bypass the reticuloendothelial system (RES) and remain in circulation time in the blood. Small size, large surface area-to-volume ratio, and molecular encapsulation capacity, along with surface functionalization, render them the most sought-after candidates for drug delivery, imaging, and therapeutic purposes (Fang et al., 2013; Steichen et al., 2013). Various types of nanoparticles have been made for biomedical applications, such as lipid-based NPs, polymeric NPs, silica NPs, and metal NPs. Lipid nanoparticles, such as liposomes, are very biocompatible and can also encapsulate hydrophilic as well as hydrophobic molecules, though they generally suffer from issues such as leakage of the content and instability, and toxicity (Sharma and Sharma, 1997; Maurer et al., 2001). To overcome these problems of immune rejection, toxicity, and biodistribution, scientists have resorted to biomimetic nanotechnology. This method applies cell membrane-coated nanoparticles (CMCNPs) that can replicate the surface characteristics of native cells. By membrane coating of nanoparticle cores from erythrocytes, leukocytes, platelets, stem cells, cancer cells, or bacteria, these devices can avoid immune detection and preserve the biological behavior of native cell membranes (Gupta et al., 2014; Zhang, 2016). CMCNPs therefore present a two-in-one platform, unifying the therapeutic adaptability of nanoparticles with the biological camouflage and targeting property of natural cells. Effective lung cancer screening following clinical trials necessitates excellent institutional support, collaboration, and multidisciplinary coordination. Administrators, radiologists, oncologists, pulmonologists, and surgeons should be engaged in program planning under a medical director who coordinates clinical, administrative, and marketing staff. Patient recruitment, supported by educational materials and outreach through print, broadcast, and electronic media to reach target smokers, is what will sustain success. Effective workflows in eligibility screening, shared decision documentation, scheduling, and billing are necessary. In the United States, USPSTF certification and CMS approval have facilitated program implementation (Moyer and Force, 2014; Richards et al., 2019). Technical characteristics involve a CT scanner of at least 16-channel and/or higher that is able to complete low-dose single-breath-hold scans and must be run by qualified CT technologists to maintain consistency and image quality. Image interpretation should ideally be conducted by thoracic radiologists or those who have experience with chest CT, and results are stored via a Picture Archiving and Communication System for use and long-term record keeping (Wolf et al., 2024).

## 9 Conclusion

Application of nanotechnology in lung cancer research has opened unique opportunities for early diagnosis and immune modulation, addressing two of the most critical challenges in oncology. Smart nanoplatforms, designed with tunable physicochemical properties and biological specificity, enable sensitive detection of circulating biomarkers such as exosomal proteins, nucleic acids, and metabolic indicators often detectable before radiographic abnormalities appear. Also, nanocarriers facilitate targeted delivery of immunomodulatory drugs, i.e., immune checkpoint inhibitors, cytokines, and tumor-associated antigens, thereby enhancing immune recognition as well as suppression reversal by tumors. Such advances hold great promise for the situation of early-stage disease, in which treatment can markedly enhance survival. Translational advances are nevertheless constrained by critical barriers despite outstanding progress. Biological heterogeneity of lung tumors, off-target accumulation, and long-term toxicity of nanomaterials continue to be challenges to reproducibility and safety. In addition, large-scale manufacturing, standardization of nanoplatform synthesis, and regulatory frameworks for nanodiagnostics and nano-immunotherapies need to be addressed urgently. The future wave of innovation will likely depend on merging nanotechnology with artificial intelligence, digital twins, and personalized medicine to forecast therapeutic outcomes and tailor treatment planning. The biodegradable nanoplatform is promising avenues for improved and safe clinical utilization. Briefly, intelligent nanoplatforms provide a paradigm shift to early diagnosis and immune modulation in lung cancer through diagnostic accuracy along with therapeutic agility. Interdisciplinary convergence between materials scientists, oncologists, and data scientists is crucial to realizing precision nanomedicine in lung cancer treatment.
